# Effect of information provision by familial nudging on attitudes toward offshore wind power

**DOI:** 10.1371/journal.pone.0297199

**Published:** 2024-01-17

**Authors:** Hidenori Komatsu, Hiromi Kubota, Kenji Asano, Yu Nagai

**Affiliations:** 1 Grid Innovation Research Laboratory, Central Research Institute of Electric Power Industry, Yokosuka-shi, Kanagawa, Japan; 2 Sustainable System Research Laboratory, Central Research Institute of Electric Power Industry, Abiko-shi, Chiba, Japan; 3 Socio-Economic Research Center, Central Research Institute of Electric Power Industry, Chiyoda-ku, Tokyo, Japan; Shandong University of Science and Technology, CHINA

## Abstract

Offshore wind power (OWP) is a promising way to achieve decarbonization and tackle global climate change, but acceptance by residents is an important issue for site location. Information provision could be a more cost-effective intervention than debates or subsidies, assuming that scientifically correct information alone is insufficient and information design to boost the message effects considering realistic human responses is necessary. Thus, we designed nudging messages to increase acceptance of OWP, using a message framework to moderate risk-averse attitudes by reminding readers of familial support based on insights from kin selection theory from evolutionary psychology. A randomized controlled trial based on an internet survey of more than 4000 responses from the general public was performed to investigate the message effects. The messages significantly moderated the risk-averse attitudes toward OWP by 0.228 on average on a five-point Likert scale, which meant that about 5 people out of 100 changed their attitudes to be safer by 1 point. This suggests that disseminating flyers using nudging messages might be an effective way to increase acceptance. We also extracted responses from those who mentioned fisheries in an open-ended question as an alternative to actual fishers. Responses from this segment were more complex and the message effects were limited compared with those who did not mention fisheries; although the attitudes toward OWP before receiving the messages were safer, reading descriptions for potential risks on fisheries may have unexpectedly led them to focus on the risks of which they were unaware at first. Because information provision based on nudging is effective but just one of a wide variety of political interventions available, practitioners should consider a combination of multiple options instead of using only nudging messages.

## Introduction

In response to the global need for decarbonization, the Japanese government set an official goal to compose a deal of 30–45 GW of offshore wind power (OWP) by 2040 [1]. OWP currently available can be categorized as fixed-bottom and floating OWP. Fixed-bottom OWP is usually installed in water up to 80 m with a monopile or a tripod base. Floating OWP is a relatively new technology for deeper water with a floating base [2]. Although the potential of fixed-bottom OWP is estimated to be 128 GW in Japan, the potential of floating OWP is estimated to be 424 GW [3], making it an important type of installation because of the limited area of shallow seas close to coasts and suitable for fixed-bottom OWP. However, local stakeholders, such as residents, tend to have negative perceptions of OWP as turbines get closer to coasts [4–7]. Moreover, floating OWP is less technically established than fixed-bottom OWP and more likely to concern stakeholders despite the larger distance from coasts, and fixed-bottom OWP installations are installed nearer coasts. Thus, acceptance by the residents of site location is important for installing wind turbines, even offshore [8], regardless of type.

There are many potential factors that affect attitudes toward OWP, such as scenery [9] and impacts on the ecology [10], economy [11], and quality of life [12]. Among these factors is subjective risk judgements by residents. The most common measures for moderating risk-averse attitudes, and hence for increasing acceptance, are debates for residents [13, 14] and subsidies (i.e., cooperation money for fisheries) [15]. These approaches are costly, and information provision may be a more promising approach that is less costly, easier to implement, and thus more scalable. Providing scientifically correct information is not always effective because human rationality is bounded [16, 17] and decision-making is not based on objective analysis of risk-benefit trade-offs [18, 19]. To tackle this problem, nudging has been used in behavioral economics as an effective intervention that works on human intuitive decision-making through small and subtle changes in choice architectures [20, 21]. For example, social comparison to show consumers how much energy they are consuming compared with others has a larger impact [22] and is a more cost-effective way [23] to promote energy conservation than highlighting economic incentives. A wide variety of applications have been proposed after the concept of nudging was introduced [21, 24], including interventions affecting attitudes toward risks, mainly related to health issues [25], and the majority used message framing, changing defaults, and social norms [26]. However, a nudging framework, even with these well-known approaches, has not been investigated for targeting OWP-related risks.

The processes of designing nudges have depended on trial and error and a consistent meta-theory has not been proposed. Given that behavioral economics emphasizes intuitive decision-making [17, 27], which could potentially have its origin in biological adaptiveness [28], nudges can be designed considering the biological adaptiveness of human psychological responses. Therefore, we have been exploring a more efficient framework for designing nudges that incorporates insights from evolutionary psychology. We constructed a multi-agent model to simulate the process of how human attitudes toward risks were evolved. The model was inspired by risk-averse behaviors, such as criticism of risk sources by emphasizing the dangers to future generations, which assumed risk aversion to be a product of altruistic behavior based on kin selection. The agents’ attitudes toward risks and strategies for altruistic behaviors to their kin agents were evolved through kin selection [29, 30]. Agents survived an environment with a tradeoff between a life-threatening risk and benefits obtained from the risk source, and the attitudes toward the risk were the target of evolution. The agents distributed the benefits to their kin agents, and the distribution strategies were another target of evolution. Agents who had more gene copies in the population were more likely to be selected as parents, and each agent shared 50% of gene copies with their parents. The parent agents reproduced offspring according to the amount of benefits they had. This cycle was repeated and the agents were evolved using a real-coded genetic algorithm [31]. Altruistic populations evolved to be more risk-averse than non-altruistic populations on average when the environment was moderate. Conversely, the altruistic populations evolved to be risk-prone on average when the environment was harsh. The most important insight obtained from this model was that there were always agents evolving to be less risk-averse who were supported by their kin agents, although the existence of kin agents in itself did not affect the attitudes. These tendencies were independent of the harshness of the environment or the averaged attitudes toward the risk of the whole populations [32]. Although these insights suggest that support from kin could affect attitudes toward risks, it is difficult to promote actual support in the real world. Thus, we sought to promote a sense of being supported by kin by using messages for information provision, assuming that reminding people of familial support would have similar effects to actual familial support. The effects of our designed messages were then investigated in the real world, with applications including moderating risk-averse attitudes toward air pollution caused by industrialization [33, 34], disposable plastics [35], and IT innovations [36]. In these previous studies, we designed the messages to highlight that the target technologies have a history of having been developed in our parents’ and grandparents’ generations and are supporting our everyday life in various ways. The studies suggested that the messages promoted a sense of receiving familial support and risk-prone attitudes, moderating the risk-averse attitudes in several countries, as our evolutionary simulation model suggested.

The present study proposes a message design using a similar framework to increase acceptance by the public of OWP by highlighting its development from onshore wind power and benefits to everyday life via infrastructure, CO_2_ reduction, and tourism, and by focusing on subjective risk judgements. Our approach uses a characteristic message design based on insights from evolutionary psychology, different from previous types of nudges. That is, the messages highlight familial support from kin via wind power by combinations of textual and illustrative information to moderate respondents’ risk-averse attitudes, which has not been investigated in the context of information provision for OWP.

Because OWP needs to be installed at sea close to coasts, OWP sites usually overlap with existing fishing grounds. Therefore, fishers who own fishing rights to these areas tend to have concerns about OWP installment. The major concerns are loss of fishing ground, decreased fishing, and displacement from the OWP site [37]. The potential benefits of OWP for fishers, such as artificial reef effects (i.e., the foundations may function as reefs to attract fish) [38] and regulations for determining appropriate distances among turbines [39], have been investigated rigorously. Hence, we summarize these measures to address the fisher’s concerns in designing our messages, while being as fair as possible and keeping the message concise.

The message effects are investigated in a randomized controlled trial (RCT) by using internet questionnaire surveys for which more than 4000 responses from the general public living in Japan were obtained. Because energy-related issues are highly likely to be affected by external factors, such as political situations or the state of society, the message effects could include other effects unrelated to the message effects of our target interventions if we just measured a simple attitude change before and after information provision. The RCT can filter out unrelated effects and measure the intervention effects of our familial nudging with a difference-in-differences (DID) estimation [40] by setting a baseline group that receives the most basic information about OWP.

Our analysis comprises three stages. First, we ascertain attitudes of the general public toward OWP under normal conditions (i.e., without any intervention). Second, we identify whether information provision based on nudging that reminds respondents of familial support can moderate risk-averse attitudes toward OWP using the whole responses. Third, we focus on those who are interested in the influence of OWP on fisheries and ascertain the message effects for this segment, compared with the other segments.

The rest of this paper is structured as follows. The settings of the experiments are detailed in the Materials and methods section. The results of experiments are presented in the Results section and discussed in the Discussion section. Finally, our concluding remarks, including directions of future work, are given in the Conclusion section.

## Materials and methods

### Survey overview

Online questionnaires were administered by a survey company in Japan that obtains samples from registered respondents. An RCT was performed to compare any effects of our nudging messages on attitudes toward OWP with those of a simpler message. Written informed consent was obtained from the respondents by the survey company. The questionnaire was fully anonymized and no personal information was collected. No samples were collected from human bodies, and it was assumed that no psychological distress would be caused to respondents. The questionnaire was approved by the ethics committee of the Central Research Institute of Electric Power Industry.

**Fig 1:**
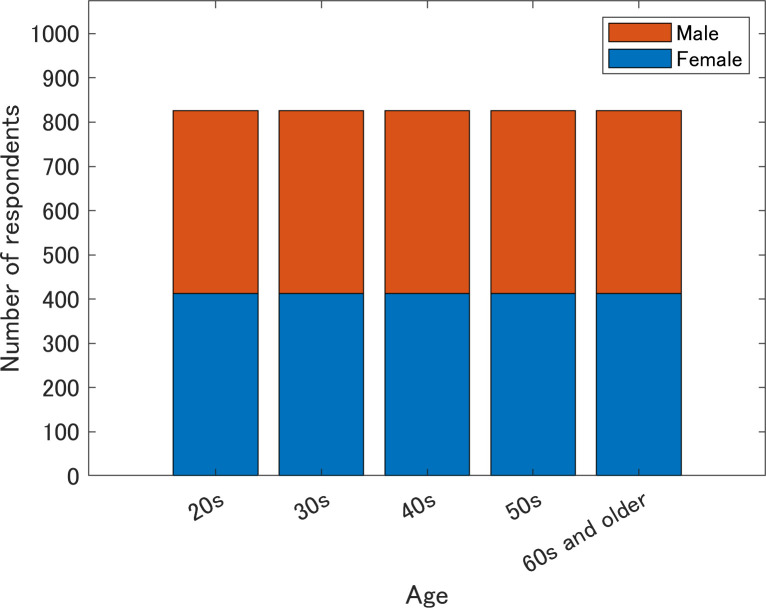
Number of respondents by age and sex.

Responses to the survey were collected between December 4 and 6, 2021. All respondents were aged 20 years and resided in Japan. Fig 1 shows the age and sex distributions of all respondents. A total of 4130 valid samples were divided into 10 equal groups based on sex and five age groups (20s, 30s, 40s, 50s, and 60s or older). Other attributes were based on incidence rate. The average age of respondents was 45.2 years (men, 45.4 years; women, 45.0 years). Because the survey company filtered out respondents who did not complete all the questions and 188 responses were excluded as a result, the 4130 responses did not include any missing values.

Respondents were first asked about their attitudes toward OWP. Then they received one of our designed messages and were asked about their attitudes toward OWP again. We ascertained the perceived support from older relatives and to younger relatives on reading the messages, as well as their thoughts via an open-ended question. Finally, we obtained attributes such as familiarity with OWP, Big Five dimensions, and fear toward COVID-19.

### Survey design for intervention

We performed an RCT using internet-based questionnaires to determine whether messages reminding people of familial support could increase positive attitudes toward OWP. This survey design of the RCT is consistent with our previous studies that identified similar message effects for topics such as industrialization [33, 34], plastic recycling [35], and IT innovations [36].

In the experiment, the respondents answered questions on how safe they think OWP is for future generations (Future generations) and for respondents themselves (Yourself), where the former is our main target variable. These responses were collected before (*Q*_pre_) and after (*Q*_post_) receiving the designed messages about OWP and were measured on a five-point Likert scale (with 1 meaning safe and 5 meaning dangerous). The 4130 respondents were stratified into 10 groups based on sex and five age groups (20s, 30s, 40s, 50s, and 60s or older). Then, the respondents in each group were randomly assigned to one of the three different conditions of information provision, that is, a control group (CG) or one of two treatment groups (T1, T2). Information provision about OWP was used as the intervention between *Q*_pre_ and *Q*_post_. Messages were designed so that the information became incrementally richer from the CG to T2; the simplest message was given to the CG, textual nudging messages were given to T1, and textual and illustrative nudging messages were given to T2. Thus, the effect of additional pieces of information should be separable (Figs A-C in S2 Appendix).

The message for CG included the simplest information and consisted of text only, which was designed to be as concise and unbiased as possible (Fig A in S2 Appendix). The descriptions referred to the advantages and disadvantages of wind power as well as the history of its development. Advantages highlighted that OWP supports a wide range of aspects of our daily life, in the form of infrastructure, CO_2_ reduction, and tourism. Disadvantages included the potential risks of collapse by natural disaster and effects on fisheries and creatures, although we also stated that these risks were being addressed with laws and technologies.

The messages for T1 included the textual information given to the CG as well as two additional pieces of text highlighting familial support (Fig B in S2 Appendix). One described the benefits of previously developed wind power as the product of previous generations and another emphasized that future OWP would support future generations.

The messages given to T2 is our main target group for this experiment (Fig C in S2 Appendix). In this treatment group, respondents were given the same information as in T1 as well as additional illustrative information. The illustration highlighted that previous generations and present generations have promoted OWP, which was developed using onshore wind power technologies, and that the benefits are received by present and future generations via infrastructure, CO_2_ reduction, and tourism. The illustrations given to respondents in T2 differed in that the ages of the people of previous and current generations in the illustrations were tailored to the generation to which the respondents belong. For example, because many respondents over the age of 50 years likely do not have living parents, in the version of illustrations given to this age bin, the people of previous generations wore traditional clothes.

In each of our previous studies [33–36], T2 showed larger intervention effects compared with T1, even though the information presented to T1 and T2 was essentially the same. Meanwhile, the CG showed the weakest intervention effects. The difference in effects between T1 and T2 was due to the illustrations provided to T2. In T1, the text provided was sometimes difficult for the respondents to interpret. The additional visual information given to T2 emphasized the supportive relationship across generations more clearly and effectively, thus increasing the sense of familial support and the intervention effects compared with T1. Based on these results, we expected to observe a similar effect in the present study for information provision about OWP.

### Statistical analysis

Responses were obtained by using the question shown in S1 Questionnaire. First, we performed analysis of variance (ANOVA) and identified that the attitudes toward OWP were homogenous within the same explained variable using pre-intervention attitudes toward OWP. Cross-tabulation analysis was performed to identify how the pre-intervention attitudes and familiarity were correlated.

The pre-intervention attitudes were compared by segment per sex and age. We then estimated the intervention effects of our nudge message by using a DID estimation, in which the effect was defined as differences in the effect size between a control group and the treatment groups. Correlation analysis was performed by the Pearson product-moment correlation coefficients between the intervention effects and the perception of benefiting from the actions of older relatives. We performed panel analysis using forced-entry linear regression. Finally, the pre-intervention attitudes were compared by segment mentioning “fisheries” or not.

In all analyses and comparisons, the Wilcoxon Rank Sum Test was employed to evaluate the disparities between the two groups, while the Wilcoxon Signed Rank Sum Test was utilized to assess variations from the zero value. All error bars in graphs were computed as 95% confidence intervals based on *t*-distributions.

## Results

### Change in attitudes by interventions

The column for *Q*_pre_ in Table 1 shows the pre-intervention attitudes toward OWP. This corresponds to how the respondents perceived OWP under normal conditions in everyday life with no intervention. One-way ANOVA revealed that the differences in the pre-intervention attitudes were statistically insignificant among the CG, T1, and T2 for both the respondents themselves (Yourself) and future generations (Future generations). These results suggest that the samples were sufficiently randomized. Within the same message group, the pre-intervention attitudes were consistently more risk-averse for Future generations than for Yourself. However, the values of *Q*_pre_ for both Yourself and Future generations were less than 3.0 on the five-point Likert scales, which corresponded to the response “Neutral” on how they perceive OWP as “Safe” or “Dangerous.” Thus, their averaged attitudes were close to “Safe” under normal conditions.

**Table 1:** Pre-intervention attitudes and attitude change toward offshore wind power with basic attributes per group. A higher value for pre-intervention attitudes indicates higher perceived danger of offshore wind power. A higher value for attitude change indicates that the perception of offshore wind power became less dangerous. CG, control group; T1, treatment group 1; T2, treatment group 2; You, Yourself; Fut, Future generations.

Group		Pre-intervention attitude (*Q*_pre_)			Attitude change (*D*)			Attributes	
		Mean	Lower end of 95% confidence interval	Upper end of 95% confidence interval	Mean	Lower end of 95% confidence interval	Upper end of 95% confidence interval	Sex ratio (males/females, %)	Mean age
CG	You	2.299	2.251	2.346	0.000	-0.034	0.034	100.0	45.1
	Fut	2.381	2.332	2.430	-0.058	-0.096	-0.019		
T1	You	2.272	2.222	2.321	0.042	0.006	0.079	100.0	45.3
	Fut	2.345	2.296	2.395	-0.007	-0.045	0.032		
T2	You	2.248	2.199	2.297	0.150	0.115	0.186	100.0	45.1
	Fut	2.337	2.288	2.386	0.172	0.130	0.213		

The difference in attitudes toward OWP between *Q*_pre_ and *Q*_post_ was defined as *D* to evaluate the DID effect of the interventions. First, we calculated the mean value of *D* for each group, and then we evaluated the DID effect by checking whether the values for T1 and T2 were larger than those for the CG. The column for *D* in Table 1 shows the difference in attitudes by message group. Yourself and Future generations had the same order of average *D* values of T2 > T1 > CG. For Yourself, the values of *D* for the CG were not significantly different from 0, which suggests that the messages provided to the CG had no statistical effect. For Future generations, however, the values of *D* for the CG were negative and this difference was significant (*p* < 0.01, *Z* = -3.03), which suggests that the message unexpectedly promoted risk-averse attitudes. T2, the main target group of this study, showed a significant increase in *D* from 0, the CG, and T1 for both Yourself and Future generations (from 0, Yourself, *p* < 0.001, *Z* = 8.21; from CG, Yourself, *p* < 0.001, *Z* = 6.46; from T1, Yourself, *p* < 0.001, *Z* = 4.29; from 0, Future generations, *p* < 0.001, *Z* = 8.17; from CG, Future generations, *p* < 0.001, *Z* = 8.52; from T1, Future generations, *p* < 0.001, *Z* = 6.42). T1 also showed an increase in *D* compared with the CG, and this difference was significant for both Yourself and Future generations (Yourself, *p* < 0.05, *Z* = 1.99; Future generations, *p* < 0.05, *Z* = 2.08). These results suggest that simply providing additional illustrative information to T2 further increased the effectiveness of the messages provided to T1 over that of the messages provided to the CG for both Yourself and Future generations. While pre-intervention attitudes were more risk-averse for Future generations than for Yourself (column *Q*_pre_ in Table 1), the post-intervention values of *D* and the DID effects were similar for both Future generations and Yourself.

**Fig 2:**
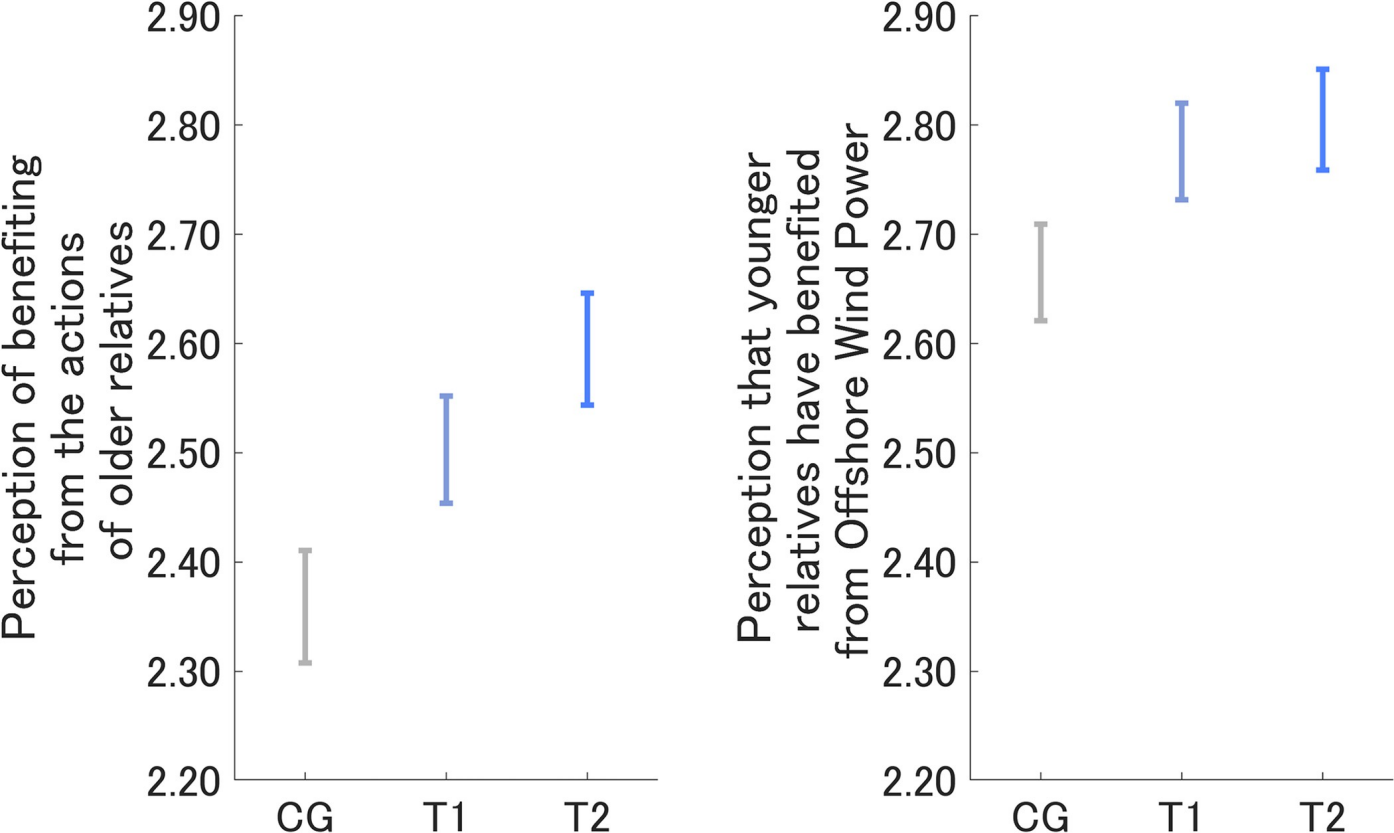
Sense of familial support from older relatives and to younger relatives on reading the designed messages by message group. Error bars show 95% confidence intervals.

During *Q*_post_, after reading the designed messages, respondents were asked how much they felt their daily life was supported by their older relatives (e.g., parents and grandparents), and the results for each group are shown on the left-hand side of Fig 2. The order was T2 > T1 > CG, and T2 and T1 were both significantly larger than the CG (T1, *p* < 0.001, *Z* = 3.96; T2, *p* < 0.001, *Z* = 6.35). In addition, respondents were asked how much they felt that the OWP would support their younger relatives (e.g., children and grandchildren), and the results for each group are shown on the right-hand side of Fig 2. The order was T2 > T1 > CG, and T1 and T2 were both significantly larger than the CG (T1, *p* < 0.001, *Z* = 3.37; T2, *p* < 0.001, *Z* = 4.45), similar to the perceived support by their older relatives.

To identify relationships between the sense of familial support and the intervention effects, we investigated the correlation of *D* for Yourself and Future generations with the two types of sense of familial support after reading the designed messages (Table 2). For the perceived support to younger relatives by OWP, the correlation coefficient in the CG was significant only for Future generations (*p* < 0.05), though the value was negative. For T1, the correlation coefficients for Yourself and Future generations were both significant (*p* < 0.001 and *p* < 0.01, respectively) for perceived support to younger relatives. The correlation coefficients for Yourself and Future generations (T1) were not significant for perceived support by older relatives. For T2, the correlation coefficient for Yourself for perceived support by older relatives was marginally significant (*p* < 0.1), while the coefficients for both Yourself and Future generations were both significant for perceived support to younger relatives (*p* < 0.001). Aggregating all three groups, the correlation coefficients were significant for Yourself for perceived support by older relatives and for both Yourself and Future generations for perceived support to younger relatives (*p* < 0.05, *p* < 0.001, and *p* < 0.01, respectively). The coefficients were smaller for perceived support by older relatives than for perceived support to younger relatives. However, the overall tendencies were T2 > T1 > CG within the same settings, only with the exception of T1 > T2 > CG for Yourself for perceived support to younger relatives. These trends were similar to our previous studies that investigated the message effects highlighting familial support to moderate risk-averse attitudes on a wide variety of topics [33–36]. The maximum values for these correlation coefficients were 0.12 [34], 0.21 [35], and 0.13 [36], respectively. The maximum value for our current study was 0.13, which was within the value range from our previous similar studies, and thus is reasonable. The results of our current study suggest that the perception of being supported by older relatives and the perception that younger relatives are being supported by OWP could increase positive attitudes, similar to previous studies on different topics.

**Table 2:** Correlation coefficients between change in attitude (*D*) and sense of familial support.

Group	Perception that respondents are being supported by older relatives		Perception that younger relatives of the respondents are being supported by offshore wind power	
	Yourself	Future generations	Yourself	Future generations
**CG + T1 + T2** **(*n* = 4130)**	*0.038	0.021	***0.095	**0.050
**CG (*n* = 1370)**	0.004	-0.021	0.044	*-0.055
**T1 (*n* = 1370)**	0.034	0.007	***0.127	**0.078
**T2 (*n* = 1390)**	‘0.049	0.037	***0.096	***0.099

‘, *, **, *** difference from zero with 90%, 95%, 99%, and 99.9% confidence, respectively.

### Pre-intervention attitudes by segment

Table 3 is a cross-table between the pre-intervention attitudes and knowledge on whether there is an on-going installment plan for OWP where they are living. For both Yourself and Future generations, the tendencies for the three types of responses are clear. If they answered “I do not know,” the numbers of those who perceived OWP as safe were decreased and the numbers who perceived OWP as dangerous increased significantly. The results for “I have heard that there is a plan” showed similar tendencies that more respondents perceived OWP as dangerous, with the only exception being that more respondents answered as “Safe” for Future generations. If they answered “I have not heard of a plan,” the opposite tendencies were observed, and more respondents perceived OWP as safe and fewer respondents perceived it to be dangerous.

**Table 3:** Cross-table between pre-intervention attitudes toward offshore wind power and knowledge on installment plans.

		Total	I have heard that there is a plan	I have not heard of a plan	I do not know
		counts			
		(4130)	363	2364	1403
Yourself	Safe	(1173)	155	^+^749	^--^269
	Slightly safe	(804)	87	^+^509	^-^208
	Neutral	(2041)	105	^-^1054	^+^882
	Slightly dangerous	(78)	^+^11	^-^40	27
	Dangerous	(34)	^+^5	^--^12	^++^17
Future generations	Safe	(1035)	^+^143	^+^652	^--^240
	Slightly safe	(832)	91	^+^520	^-^221
	Neutral	(2069)	105	1095	^+^869
	Slightly dangerous	(152)	15	^-^78	59
	Dangerous	(42)	^++^9	^--^19	14

Odds ratio: ^++^, + 10%; ^+^, + 5%; ^-^, - 5%; --, -10%.

Table 4 shows a cross-table between the pre-intervention attitudes and distances from the respondents’ houses to the nearest coast. The results showed a similar tendency to Table 3. The respondents who answered “I do not know” perceived OWP as more dangerous. For other respondents who gave a distance, the respondents perceived OWP as safer as the distance increased. These tendencies were similar for both Yourself and Future generations.

**Table 4:** Cross-table between pre-intervention attitudes toward offshore wind power and the distance from the respondent’s house to the nearest coast.

		Total	0–1 km	1–3 km	3–5 km	5–10 km	10–20 km	20 km or longer	I do not know
		Counts							
		(4130)	125	221	293	374	396	1528	1193
Yourself	Safe	(1173)	39	79	97	132	144	^+^521	^--^161
	Slightly safe	(804)	26	46	78	96	89	^+^341	^--^128
	Neutral	(2041)	50	88	112	142	158	^-^629	^++^862
	Slightly dangerous	(78)	^+^8	8	5	^-^2	^-^3	30	22
	Dangerous	(34)	2	^-^0	1	2	2	^--^7	^++^20
Future generations	Safe	(1035)	34	73	83	121	123	^+^443	^--^158
	Slightly safe	(832)	28	40	78	97	100	^+^360	^--^129
	Neutral	(2069)	56	97	112	143	161	^-^659	^++^841
	Slightly dangerous	(152)	5	6	16	12	8	58	47
	Dangerous	(42)	2	^+^5	4	^-^1	4	^--^8	^++^18

Odds ratio: ^++^, + 10%; ^+^, + 5%; ^-^, - 5%; --, -10%.

The two cross-tables (Tables 3 and 4) show that the respondents were more risk-averse toward OWP if they did not know how the technologies could affect them or if they knew that OWP could be installed nearby. Conversely, they perceived it as safer when they knew that their life would not be affected.

**Fig 3:**
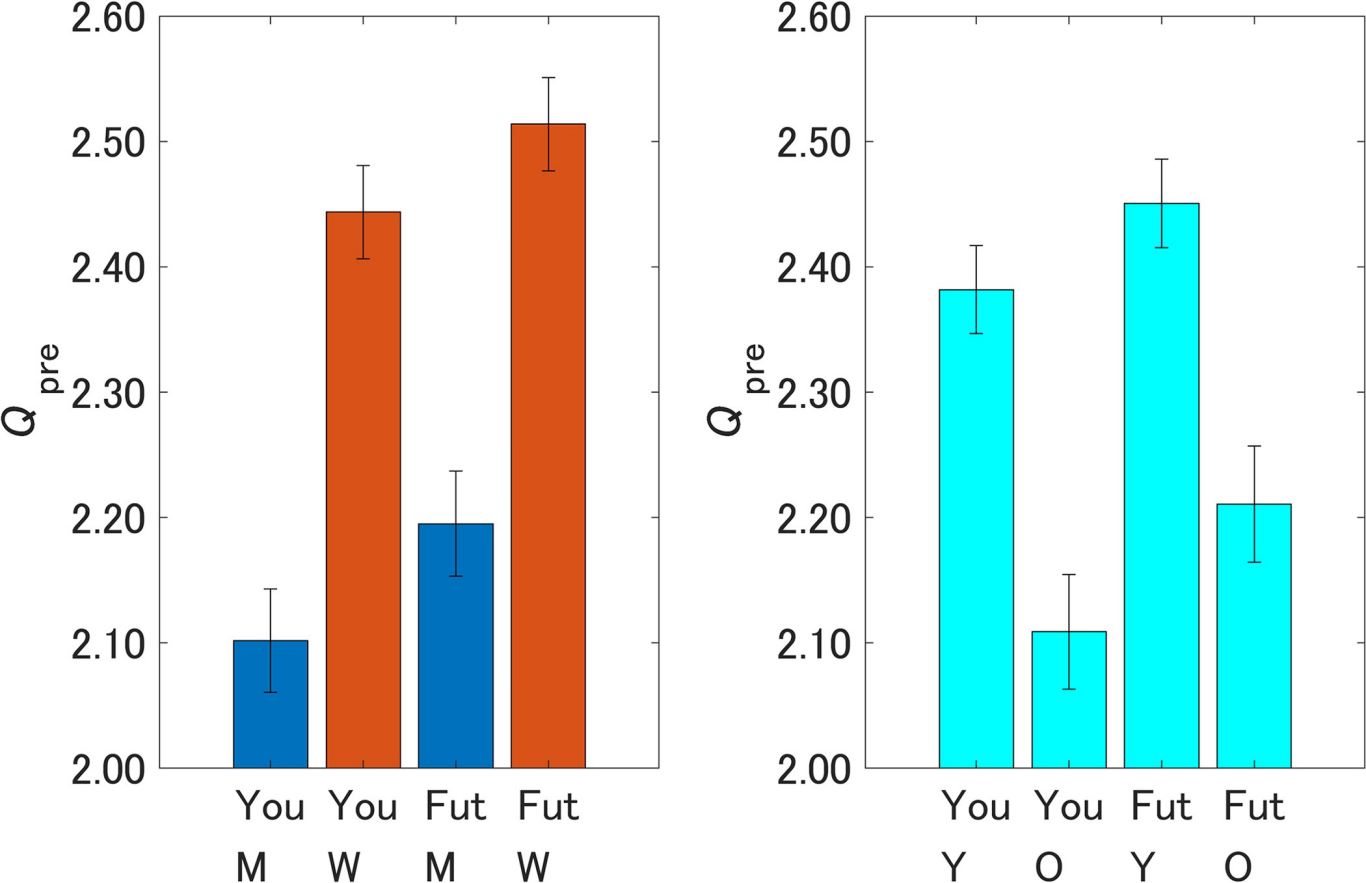
Pre-intervention attitudes toward offshore wind power by sex and age. You, Yourself; Fut, Future generations; M, men; W, women; Y, respondents under 50 years old; O, respondents over 50 years old. A higher value on the vertical axis indicates higher perceived danger of offshore wind power. Error bars show 95% confidence intervals.

The left-hand side of Fig 3 shows the pre-intervention attitudes toward OWP according to sex. Similar to the results for *Q*_pre_ in Table 1, both men and women showed more risk-averse attitudes for Future generations than for Yourself, and this difference was significant within each sex (men, *p* < 0.01, *Z* = 3.01; women, *p* < 0.05, *Z* = 2.47). Compared with men, women showed more risk-averse attitudes within the same explained variable (Yourself or Future generations).

The right-hand side of Fig 3 shows the pre-intervention attitudes toward OWP by age, where age bins were aggregated into two bins for under 50 years old and over 50 years old. Both age groups showed more risk-averse attitudes for Future generations than for Yourself, similar to those for *Q*_pre_ in Table 1. For both Yourself and Future generations, younger respondents showed significantly more risk-averse attitudes than older respondents (under 50 years old, *p* < 0.001, *Z* = 9.48; over 50 years old, *p* < 0.001, *Z* = 8.37).

### Intervention effects by segment

**Fig 4:**
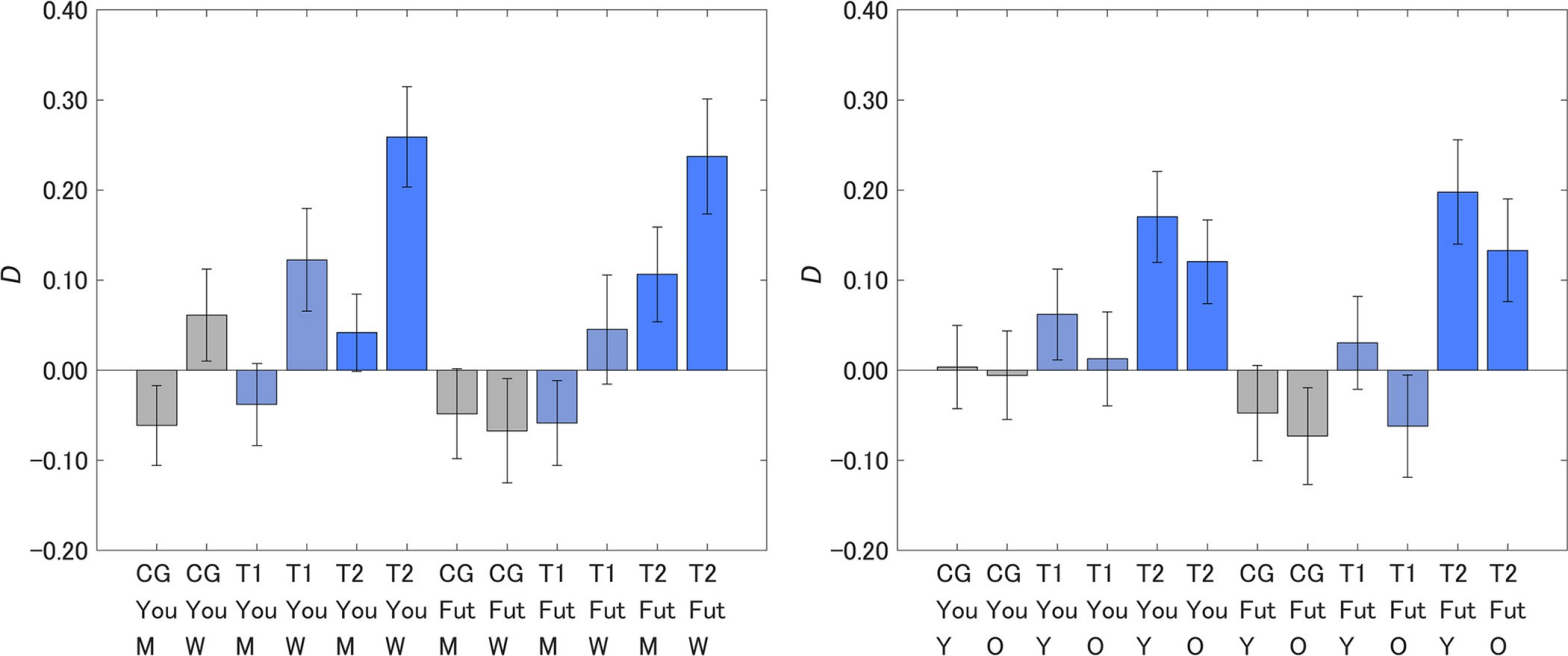
Attitude change toward offshore wind power after receiving a designed message (*D*) by sex and age. A higher value on the vertical axis indicates lower perceived danger of offshore wind power. You, Yourself; Fut, Future generations; M, men; W, women; Y, respondents under 50 years old; O, respondents over 50 years old. Error bars show 95% confidence intervals.

Responses were divided by sex to identify any differences in the message effects (left-hand side of Fig 4). The difference was not significant only for CG for Future generations, though women showed larger message effects compared with men in all the other groups (CG, Yourself, *p* < 0.001, *Z* = 4.07; T1, Yourself, *p* < 0.001, *Z* = 4.46; T2, Yourself, *p* < 0.001, *Z* = 6.08; T1, Future generations, *p* < 0.01, *Z* = 3.10; T2, Future generations, *p* < 0.001, *Z* = 3.89). The intervention effect was largest for women in T2. Within the same sex group, values of *D* were in the order T2 > T1 > CG, with the only exception being men for Future generations. This suggests that the size of intervention effects was in the order T2 > T1 > CG, independent of sex, which is similar to the aggregated results for both Yourself and Future generations (column *D* in Table 1). Samples were also divided by age into groups of younger (age under 50 years) and older (age 50 years or older) respondents (right-hand side of Fig 4). Within the same age group, values of *D* were in the order T2 > T1 > CG. This suggests that the size of intervention effects was in the order T2 > T1 > CG, independent of whether the respondents were over 50 years old, which is similar to the aggregated results having the same order for both Yourself and Future generations (column *D* in Table 1).

### Panel data analysis

To identify the DID effects of T2 messages and the other effects of respondents’ attributes, the following two regression models, Eqs (1) and (2), were constructed. The personality attributes were sampled using the short Japanese version of the Big Five model [41]. *COVID_fe_* was fear toward COVID-19, measured by a seven-item scale [42]. *COVID_pr_* measured how much respondents were preoccupied with COVID-19. These variables were introduced because we found that attitudes to COVID-19 could influence the intervention effects for other topics [34, 35]. *OWP_se_* was a dummy variable to identify whether respondents have seen OWP before or not, considering that familiarity with the risk source could moderate risk-averse attitudes [43]. *F* was a dummy variable to identify whether respondents mentioned fisheries in an open-ended question. The questions for *COVID_pr_*, *OWP_se_*, and *F* are described in the Supplemental materials.


$$D_{y}=a_{1}\times T1+a_{2}\times T2+a_{3}\times S+a_{4}\times A+a_{5}\times P_{op}+a_{6}\times P_{co}+a_{7}\times P_{ex}+a_{8}\times P_{ag}+a_{9}\times P_{ne}+a_{10}\times {COVID}_{fe}+a_{11}\times {COVID}_{pr}+a_{12}\times {OWP}_{se}+a_{13}\times F+a_{14}$$ (1)

$$D_{f}=a_{1}\times T1+a_{2}\times T2+a_{3}\times S+a_{4}\times A+a_{5}\times P_{op}+a_{6}\times P_{co}+a_{7}\times P_{ex}+a_{8}\times P_{ag}+a_{9}\times P_{ne}+a_{10}\times {COVID}_{fe}+a_{11}\times {COVID}_{pr}+a_{12}\times {OWP}_{se}+a_{13}\times F+a_{14}$$ (2)

*T*1, *T*2: Target of the intervention in T1 and T2, respectively (0: no, 1: yes)

*S*: Sex (0: men; 1: women)

*A*: Age

*P_op_*: Openness

*P_co_*: Conscientiousness

*P_ex_*: Extraversion

*P_ag_*: Agreeableness

*P_ne_*: Neuroticism

*COVID_fe_*: Fear toward COVID-19

*COVID_pr_*: Preoccupied with COVID-19

*OWP_se_*: Have seen offshore wind power before (0: no, 1: yes)

*F*: Mentioned fisheries (0: no, 1: yes)

*a_1_*–*a_13_*: Coefficients for each term

*a_14_*: Intercept

Here, *D_y_* is *D* for Yourself and *D_f_* is *D* for Future generations. Coefficients *a_1_*–*a_13_* and intercept *a_14_* were determined using forced-entry regression (Table 5). *T2* for Yourself was estimated as 0.149, and *T2* for Future generations, the main target variable, was estimated as 0.228. This suggests that our designed message for T2 effectively increased positive attitudes toward OWP, as we had intended. Estimates for T1 were positive and marginally significant. Overall, these results suggest that the intervention for T2, which included additional text and illustrations, was the largest of the three message groups. Both estimates for sex were positive and significant, suggesting that being a woman contributed to the message effects. Age showed significant negative effects, suggesting that younger respondents contributed to the message effects. For the personality variables, openness and agreeableness had significant positive effects for Yourself, and extraversion had significant positive effects for Future generations. Fear toward COVID-19 showed significant positive effects for Future generations, although being preoccupied with COVID-19 showed negative effects for both Yourself and Future generations. Having seen OWP before had positive effects for both Yourself and Future generations. Mentioning fisheries in the open-ended questionnaire had significant negative effects for both Yourself and Future generations. The open-ended question was provided after the respondents received our designed messages. Thus, one possible explanation for our results is that the respondents may have first been made aware of the risks from the messages and have come to perceive OWP as more dangerous, although they had originally perceived OWP as safe. The negative effects of mentioning fisheries are analyzed in more detail in the next section.

**Table 5:** Coefficients from linear regression analysis.

Explained variables		Yourself			Future generations		
		Estimated coefficients	SE	*t*	Estimated coefficients	SE	*t*
**Intercept**		**-0.417	0.132	-3.165	*-0.295	0.148	-1.995
**Intervention**	T1	‘0.044	0.025	1.720	‘0.052	0.028	1.823
	T2	***0.149	0.025	5.906	***0.228	0.028	8.074
**Attribute variables**	Sex	***0.144	0.021	6.724	*0.060	0.024	2.521
	(Men = 0, Women = 1)						
	Age	**-0.002	0.001	-2.956	***-0.003	0.001	-3.684
**Personality variables**	Openness	*0.004	0.004	0.806	0.013	0.005	2.576
	Conscientiousness	0.015	0.005	2.849	0.001	0.006	0.110
	Extraversion	0.003	0.005	0.685	*0.007	0.006	1.277
	Agreeableness	**0.004	0.005	0.786	-0.004	0.006	-0.616
	Neuroticism	-0.012	0.005	-2.438	-0.007	0.006	-1.206
**Other variables**	Fear toward COVID-19	0.003	0.002	1.384	**0.008	0.003	3.036
	Preoccupied with COVID-19	**-0.045	0.014	-3.264	‘-0.026	0.015	-1.713
	Have seen offshore wind power before	**0.048	0.015	3.159	‘0.029	0.017	1.712
	(0: no, 1: yes)						
	Mentioned fisheries	*-0.138	0.069	-2.004	**-0.224	0.077	-2.899
	(0: no, 1: yes)						
**Root Mean Squared Error**		0.662			0.742		
**Adjusted *R*** ^**2**^		0.033			0.027		
***F*-statistic vs. constant model**		11.9			9.78		
***p*-value**		< 0.001			< 0.001		
**Error degrees of freedom**		4116			4116		
**Number of valid samples**		4130			4130		

‘, *, **, *** difference from zero with 90%, 95%, 99%, and 99.9% confidence, respectively.

### Respondents showing interest about fisheries

Fishers are one of the most important stakeholders for the site location of OWP because their approval is necessary when the wind turbines can affect their fishing sites [44–46]. Therefore, how fishers feel about the information provision must be identified. However, it is difficult to obtain enough responses from fishers due to the diminishing numbers of people engaged in fisheries in Japan [47]. Thus, instead of obtaining responses from fishers, we extracted respondents who mentioned “fisheries” in an open-ended question that ascertained what they felt on reading our designed messages, and we assumed that these respondents are highly interested in fisheries. There were 95 of these valid respondents out of the whole sample of 4130, which was only 2.3% of respondents; thus, these samples may not be representative of a certain group or those who really were interested in fisheries, and thus the universality of the insights might be limited. However, analysis of those who showed interest in fisheries could be beneficial to predict actual fishers’ perception as a proxy variable for actual fishers, assuming the limitation of the statistical power.

**Fig 5:**
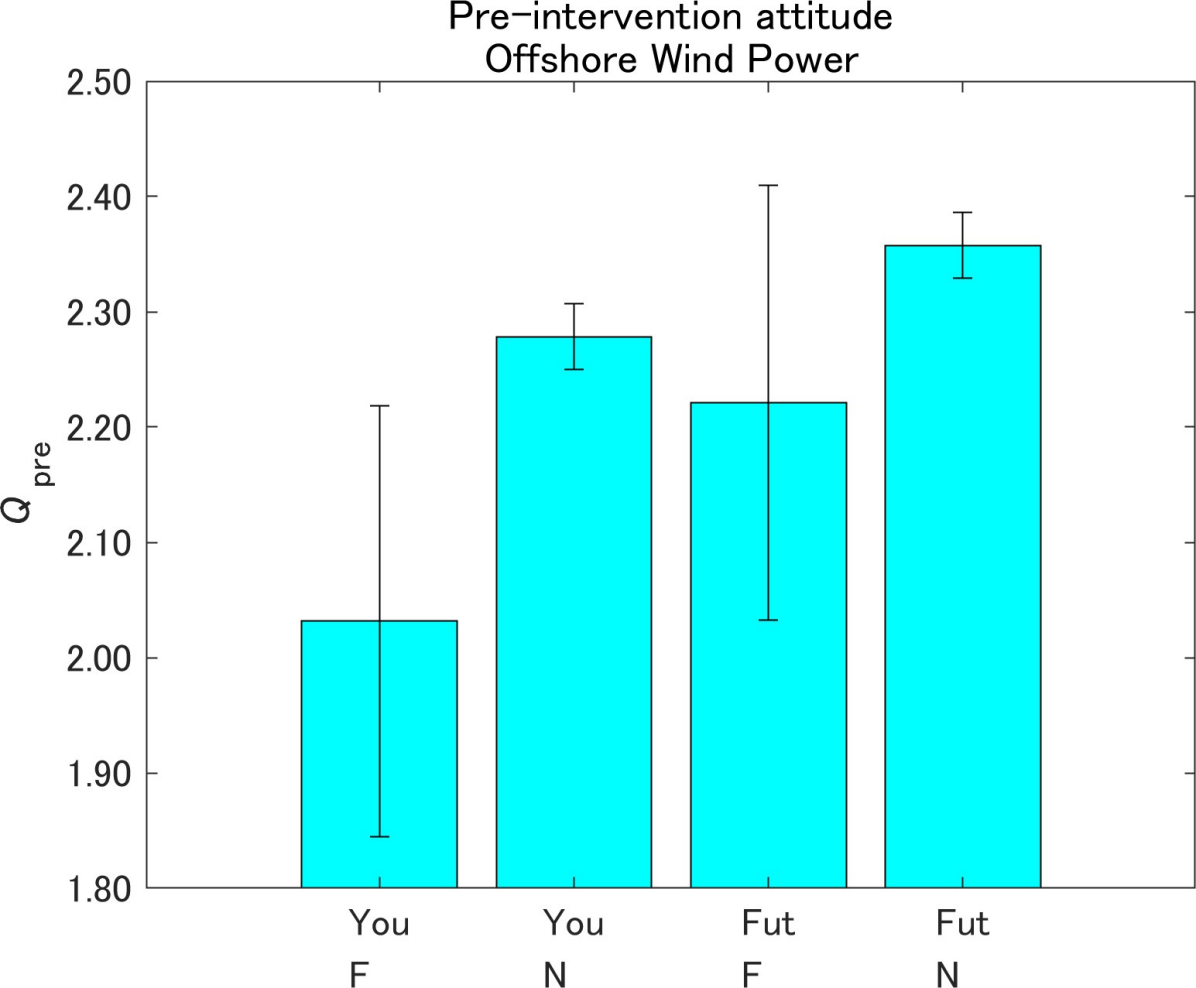
Pre-intervention attitudes toward offshore wind power for the group who mentioned “fisheries” and the group who did not. A higher value on the vertical axis indicates more perceived danger of offshore wind power. You, Yourself; Fut, Future generations; F, respondents who mentioned “fisheries” in the open-ended question; N, respondents who did not mention “fisheries” in the open-ended question. Error bars show 95% confidence intervals.

The average age of these respondents was 45.7, which was similar to the average age of the whole sample. The number of men was 31 and women was 64, suggesting a high ratio of female respondents. Fig 5 shows the pre-intervention attitudes toward OWP for Yourself and Future generations, dividing the responses from those who mentioned “fisheries” (F) and who did not (N). Although the error bars were large for both Yourself and Future generations due to the limited number of responses, those who mentioned “fisheries” perceived OWP as safer than those who did not. The difference was significant, especially for Yourself (*p* < 0.05, *Z* = 2.51).

We categorized all the 95 respondents in the open-ended question who mentioned “fisheries” in the open-ended question (F in Fig 5) manually into each of the three groups (Negative, Neutral, and Positive) in terms of perception on how fisheries could be affected. Although this categorization cannot be totally objective, there were 13 neutral responses, which were neither negative nor positive, and 7 out of these 13 responses stated that they were willing to learn about OWP. The rest of 82 responses showed interest in the effects of OWP. Thus, the majority of the 95 responses that referred to “fisheries” did not do so incidentally. There were 9 positive responses and 73 negative responses, where most of the responses were negative and mentioned that they were anxious about the effects on fisheries. The positive responses suggested that they would accept OWP if it were installed properly, or they simply value the benefits more than the risks, although these types of responses were least frequent. We also observed responses corresponding to the explanation that receiving our designed messages brought the risks of the effects of OWP on fisheries to their attention. For example, the second and third responses shown in the Negative column of Table B in S1 Appendix suggest that reading the messages caused them to perceive OWP as dangerous.

## Discussion

The present study investigated how respondents perceived OWP as safe or dangerous before receiving any information under normal conditions. For both Yourself and Future generations, younger respondents tended to perceive OWP as more dangerous. They became risk-averse when OWP was, or might be, relevant to areas where they were living, whereas their perception was safer when OWP was irrelevant to them. These tendencies suggest that site location of OWP is a “not in my back yard” problem [9, 48], which is similar to responses on onshore wind power where the acceptance changes greatly depending on whether they are residents [49]. The perception of respondents with no knowledge about OWP was dangerous, which corresponds to well-known responses toward unknown risks [42, 50–52]. We measured the attitudes toward OWP with five-point Likert scales (1: Safe, 3: Neutral, 5: Dangerous). The average responses for both Yourself and Future generations were lower than Neutral. Thus, the attitudes can be judged as relatively safe, compared with air pollution caused by industrialization [34] or disposable plastics [35]. Despite these overall tendencies to perceive OWP as safe, the perceptions for Future generations were significantly more dangerous than for Yourself, suggesting potential anxiety for the future of the technologies.

Next, we investigated the effects on moderate risk-averse attitudes toward OWP of messages that reminded respondents of familial support. The size of the message effects was in the order T2 > T1 > CG, which corresponded to the order of the sense of familial support from older relatives and to future generations on reading the messages. This suggests that our designed message increased the sense of familial support and moderated risk-averse attitudes toward OWP as we intended. The larger increase in the perceived familial support by older relatives than that in the perceived support of future generations in the current study was similar to all our previous studies on multiple topics for information provision [33–36]. Although the DID effects for Future generations were consistently significant in all our previous studies, the effect sizes for Yourself were weaker in some cases [33, 35] than others [34, 36]. In contrast, the current study showed significant DID effects for both Yourself and Future generations, even though the pre-intervention attitudes were more risk-averse for Future generations than for Yourself. The degree of the message effect, especially for T2 for Future generations as our main target group, was 0.228 on the five-point Likert scale and was significantly different from the CG when segregating all the other effects by basic attributes. This effect size corresponded to about 5 respondents out of 100 changing their attitudes to be safer by 1 point. The adjusted *R*^2^ values of 0.033 for Yourself and 0.027 for Future Generations are reasonable, considering the values from a similar type of experiments [33–36] as well as RCT experiments on nudging information provision using social norms [22].

For the other attributes in our previous experiments, being a woman showed a significantly larger message effect in some cases [33–35], whereas smaller sex differences were observed in another case [36]. The current study was similar to these previous studies, and the sex differences in the responses to the messages were clearer compared with the previous studies. Younger respondents showed a larger significant increase in the message effect in some of our previous experiments [33, 34], although the effect sizes were sometimes insignificant [36] or the effect was even negative [35]. In the current study, being young consistently contributed to larger message effects for all message groups. For the personality traits, agreeableness showed significant positive contributions only for Yourself. Although agreeableness showed consistently significant positive contributions for both Yourself and Future generations in our previous studies on information provision for environmental issues [33–35], the effects were weak in the present study. Openness for Yourself and extraversion for Future generations showed significant positive contributions too. These two variables were partly significant [35] or sometimes showed negative contributions [34] in our previous studies, and appear to be strongly case-sensitive. For other COVID-19-related variables, the fear toward COVID-19 was positive and significant only for Future generations, which was the opposite of the results for information provision on disposable plastics [35]. Being preoccupied with COVID-19 showed greater negative effects for both Yourself and Future generations. These tendencies are consistent with the finite pool of worry hypothesis [53, 54]. Because COVID-19 was still a great social problem in Japan during 2021, for those who were afraid of COVID-19 and did not have mental space to worry about anything else, OWP may not have been a target of concern, and thus the information provision may have been ineffective. Having seen OWP before showed significant positive effects on the information provision for both Yourself and Future generations, which corresponds to the well-known effect that familiarity with risk sources moderates risk-averse attitudes [51].

Because a nudging approach is less costly than other types of interventions using economic incentives, information provision based on nudges is generally cost-effective [22, 23]. Flyers or posters could be efficient ways to disseminate our designed messages and to promote OWP. However, although the size of the effect of our familial nudging is significant, it is also small. Message design should not be considered as the sole intervention but one option in a wide variety of possible political measures. Thus, practitioners of political interventions should consider a combination of multiple options rather than nudging messages alone.

The limitation of our current approach is the intervention effects for those who are concerned about influence on fisheries. Although the number of responses were limited, mentioning fisheries in the open-ended question showed clear negative contributions for the information provision. Although those who mentioned fisheries were less risk-averse toward OWP than those who did not before receiving the information, the message effects were lower for those who mentioned fisheries. Because our designed messages described the potential risks to fisheries, one possible interpretation is that these respondents may be aware of the risks and reading the designed messages made them more risk-averse. In addition, most of the responses in the open-ended question showed negative attitudes toward OWP, some of which suggested that the respondents were close to fisheries. These results suggest that using information provision to increase acceptance of OWP by fishers, who are major stakeholders for the site locations, would be more difficult than increasing acceptance by the general public.

## Conclusion

The message effects of familial nudging were identified by an RCT using internet-based questionnaires with more than 4000 responses from the general public in Japan. The intervention effect was positive and significant for perceived risks affecting future generations, which yielded an increase of 1 point on a five-point Likert scale by 5 respondents out of 100 on average.

Although nudging is generally a cost-effective intervention depending on how the information is presented, the effect sizes of our approach are small but significant, similar to other types of information provision based on nudging, which cannot be expected to change attitudes toward the technologies drastically. This nudging approach should be used with multiple interventions, not alone.

A limitation of our study is that the tendencies observed in those who mentioned “fisheries” may not be universal due to the limited number of the respondents. The effect sizes of our designed message were decreased for this segment. These tendencies, however, could be within statistical fluctuations and might not be reproducible, especially for actual fishers. In future work, more responses will be needed for stronger statistical robustness, as well as validation for applying our message framework for information provision targeting fishers.

## Supporting information

S1 Questionnaire (DOC)

S1 Appendix (DOC)

S2 Appendix (DOC)
