# Supplementary material for: Effect of information provision by familial nudging on attitudes toward offshore wind power
Source: PLoS One. 2024 Jan 17;19(1):e0297199. doi: 10.1371/journal.pone.0297199 (PMC10793903; doi:10.1371/journal.pone.0297199)
Supplement: S1 Questionnaire — (DOC) [file pone.0297199.s001.doc]

**S1 Questionnaire**

**Questions for interventions**

- *Q*_pre_

Please answer questions about “offshore wind power.”

What do you think of the impact of offshore wind power on the following people?

|  | **Safe** | **Slightly safe** | **Neutral** | **Slightly dangerous** | **Dangerous** |
| --- | --- | --- | --- | --- | --- |
| **Future generations** | 1 | 2 | 3 | 4 | 5 |
| **Yourself** | 1 | 2 | 3 | 4 | 5 |

- *Q*_post_

(One of the messages for CG, T1, and T2 is shown.**)**

Please answer the question below again. What do you think of the impact of offshore wind power on the following people?

|  | **Safe** | **Slightly safe** | **Neutral** | **Slightly dangerous** | **Dangerous** |
| --- | --- | --- | --- | --- | --- |
| **Future generations** | 1 | 2 | 3 | 4 | 5 |

**Other questions**

- *Q*1

(One of the messages for CG, T1, and T2 is shown.**)**

Upon reading the passage above, do you think you are receiving benefits that increase your quality of everyday life from the following people?

(Your older relatives, including parents or grandparents)

| **I am benefitting** | **I am benefitting slightly** | **I am not benefitting much** | **I am not**  **benefitting** |
| --- | --- | --- | --- |
| 1 | 2 | 3 | 4 |

- *Q*2

(One of the messages for CG, T1, and T2 is shown.**)**

Upon reading the passage above, do you think offshore wind power is giving the benefits of increased quality of everyday life to the following people?

(Your younger relatives, including children or grandchildren)

| **It has benefits** | **It has some benefits** | **It has few benefits** | **It does not**  **have benefits** |
| --- | --- | --- | --- |
| 1 | 2 | 3 | 4 |

- *Q*3

(One of the messages for CG, T1, and T2 is shown.**)**

Please let us know your impressions or thoughts when you read the passage above. Please answer freely and as precisely as possible.

- *Q*4

Have you ever seen “offshore wind power” before?

1. I have never seen it
2. I have seen it on TV, newspapers, or the internet etc. (but never seen it in real life)
3. I have seen it in real life

- *Q*5

Do you know whether there is a plan for installing “offshore wind power” around your local or familiar coasts?

1. I have heard that there is a plan
2. I have not heard of a plan
3. I do not know

- *Q*6

Please choose the direct distance from your house to the nearest coast.

* If you are unsure about the exact distance, please choose one that you think describes best.

* If you have multiple houses, please choose the one for your main house.

1. 0–1 km
2. 1–3 km
3. 3–5 km
4. 5–10 km
5. 10–20 km
6. 20 km or longer
7. I do not know

- *Q*7

Since its onset, how has COVID-19 made you feel? Please select the answer that applies the most to you for each of the following items.

(I am preoccupied with COVID-19.)

| **Very characteristic** | **Characteristic** | **Neutral** | **Uncharacteristic** | **Very uncharacteristic** |
| --- | --- | --- | --- | --- |
| 1 | 2 | 3 | 4 | 5 |
