# Supplementary material for: Effect of information provision by familial nudging on attitudes toward offshore wind power
Source: PLoS One. 2024 Jan 17;19(1):e0297199. doi: 10.1371/journal.pone.0297199 (PMC10793903; doi:10.1371/journal.pone.0297199)
Supplement: S1 Appendix — (DOC) [file pone.0297199.s002.doc]

**S2 Appendix**

**Tables**

Table A shows the numbers of collected samples by prefecture. Prefectures with sample counts larger than 100 and amount of fish caught larger than 100 kt were extracted from the 47 prefectures in Japan. Because we collected responses based on incident rate, except for age and sex, the counts for each prefecture were proportional to the prefectures’ population. The amount of fish caught is also shown for each prefecture to indicate which prefectures have active fisheries. Saitama is an inland prefecture, and thus the amount of fish caught is not counted.

**Table A. Number of Collected Samples and Amount of Fish Caught by Prefecture**

| **Prefecture** | **Collected samples** | | **Amount of fish caught** | |
| --- | --- | --- | --- | --- |
|  | **Counts** | **Percentage** | **Weight (kt)** | **Percentage** |
| **Hokkaido** | 212 | 5.1 | 910.4 | 28.5 |
| **Miyagi** | 72 | 1.7 | 161.5 | 5.1 |
| **Ibaraki** | 76 | 1.8 | 299.4 | 9.4 |
| **Saitama** | 249 | 6.0 | 0.0 | 0.0 |
| **Chiba** | 226 | 5.5 | 105.4 | 3.3 |
| **Tokyo** | 537 | 13.0 | 29.0 | 0.9 |
| **Kanagawa** | 348 | 8.4 | 26.2 | 0.8 |
| **Shizuoka** | 112 | 2.7 | 243.8 | 7.6 |
| **Aichi** | 274 | 6.6 | 52.8 | 1.7 |
| **Mie** | 55 | 1.3 | 107.2 | 3.4 |
| **Osaka** | 340 | 8.2 | 17.9 | 0.6 |
| **Hyogo** | 224 | 5.4 | 48.3 | 1.5 |
| **Fukuoka** | 158 | 3.8 | 23.9 | 0.7 |
| **Nagasaki** | 28 | 0.7 | 242.7 | 7.6 |
| **Miyazaki** | 18 | 0.4 | 101.0 | 3.2 |

Prefectures with sample counts larger than 100 and amount of fish caught larger than 100 kt were extracted from the 47 prefectures in Japan. Based on data retrieved from https://www.e-stat.go.jp/stat-search/files?stat_infid=000032205422.

Table B shows examples of characteristic responses from the 95 respondents.

**Table B. Characteristic Responses from Respondents Who Mentioned “Fisheries” in the Open-ended Question**

| **Negative** | - *Please do not trouble fishers.* (Man in his 40s, *D* = -1 for Yourself, -1 for Future generations, living in Kagawa, CG.) - *I often see wind power in the inner sea of Hokkaido recently and have been thinking that wind power is ideal. But I admit that they could collapse and might be dangerous, which made me startled. My parents have been working as fishers until recently. Fishing would be harder if they had to pass by wind turbines. Maybe marine creatures would disappear. Though I was just thinking that wind power is clean, I started to think that it might not only ruin scenery but also affect life of people and creatures.* (Woman in her 50s, *D* = 1 for Yourself, -2 for Future generations, living in Hokkaido, CG.) - *I wanted to push wind power for nuclear power phase-out, but I realized that wind power has its own problems too. I wish that the facilities will be more compact with less risks of collapse and bird strikes, and have less impact on fishing sites and marine creatures.* (Woman in her 40s, *D* = -1 for Yourself, -2 for Future generations, living in Saitama, T1.) |
| --- | --- |
| **Neutral** | - *I wondered what the influence on fisheries and marine creatures exactly meant.* (Woman in her 30s, *D* = 1 for Yourself, -1 for Future generations, living in Kanagawa, T2.) - *I want to know the details on how much and in what way risks of the collapse and the influence on fisheries and marine creatures really are.* (Woman in her 30s, *D* = 0 for Yourself, 0 for Future generations, living in Chiba, CG.) - *I was not clear with why the development of wind power affected fisheries.* (Woman in her 30s, *D* = 0 for Yourself, 0 for Future generations, living in Kanagawa, T2.) |
| **Positive** | - *Although contributions to infrastructure and measures for climate change sometimes contradict to influence on fisheries etc., I think benefits exceed the risks in total. Japan is a country with limited resources and what is usable should be used as much as possible. It is natural that the risks increase but I want to believe that we can decrease the risks in the end as a country of technologies….* (Man in his 70s, *D* = 1 for Yourself, 0 for Future generations, living in Hokkaido, T2.) - *Though I am very concerned about the collapse and bird strikes etc. in the sea, we can choose the site to avoid the problems on scenery and fisheries. My conclusion is that we should install them rigorously on the assumption that efficient installing methods are being developed.* (Man in his 60s, *D* = 0 for Yourself, 0 for Future generations, living in Aichi.) - *Though there are risks of the collapse by natural disasters such as typhoons, scenery change, influence on fisheries and marine creatures, and bird strikes, I thought offshore wind power should be promoted more because of the large contributions to CO_2_ reduction.* (Man in his 50s, *D* = 0 for Yourself, 0 for Future generations, living in Hyogo.) |
