# Supplementary material for: Effect of information provision by familial nudging on attitudes toward offshore wind power
Source: PLoS One. 2024 Jan 17;19(1):e0297199. doi: 10.1371/journal.pone.0297199 (PMC10793903; doi:10.1371/journal.pone.0297199)
Supplement: S2 Appendix — (DOC) [file pone.0297199.s003.doc]

**S3 Appendix**

**Figures**

Fig A, B, and C show the messages presented to CG, T1, T2, respectively.


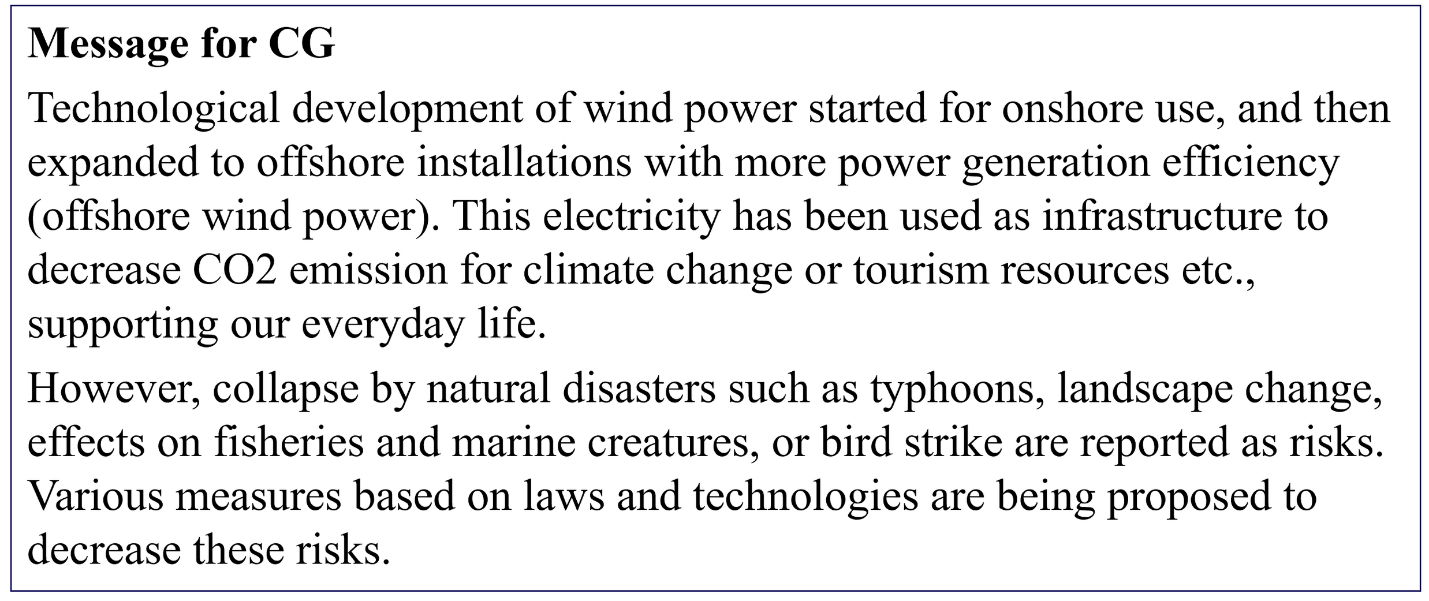


**Fig A. Message Presented to the CG Group.**


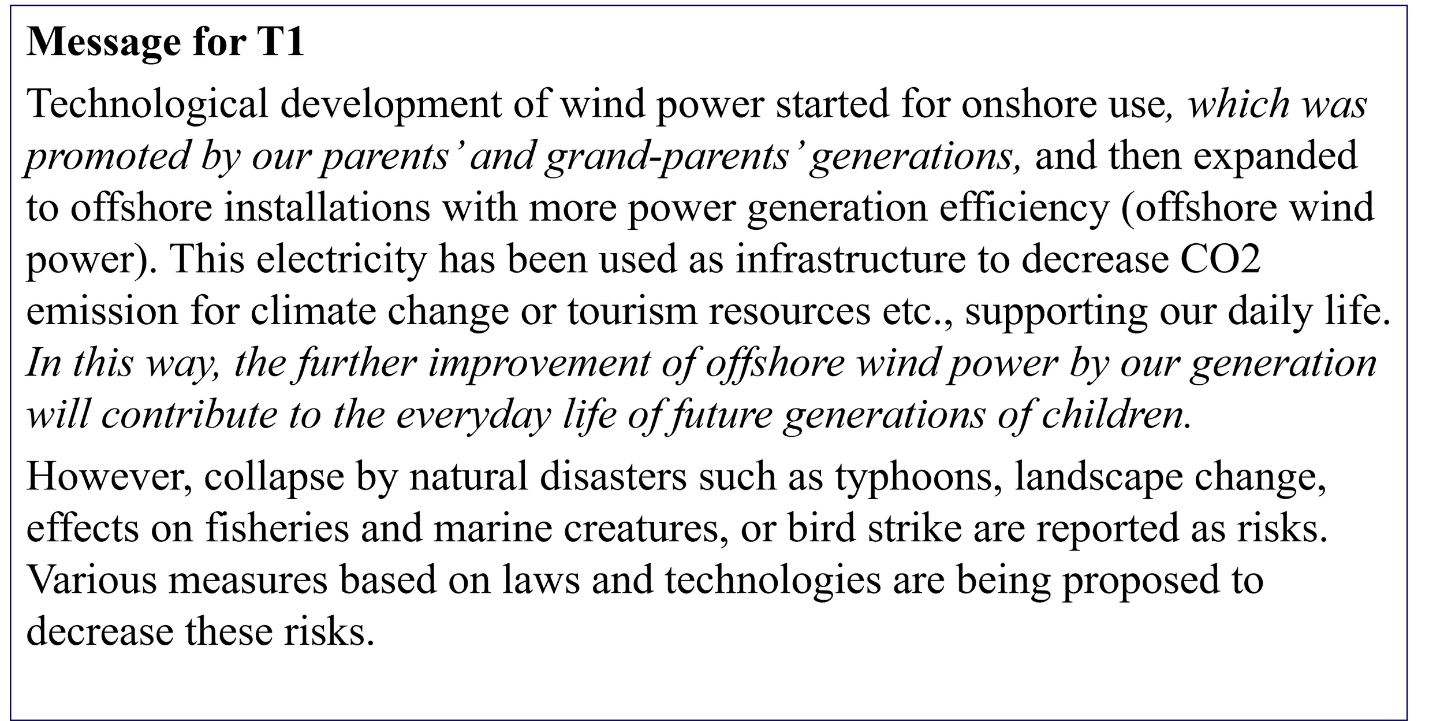


**Fig B. Messages Presented to the T1 Group.** Italic parts in the text are the intervention messages.


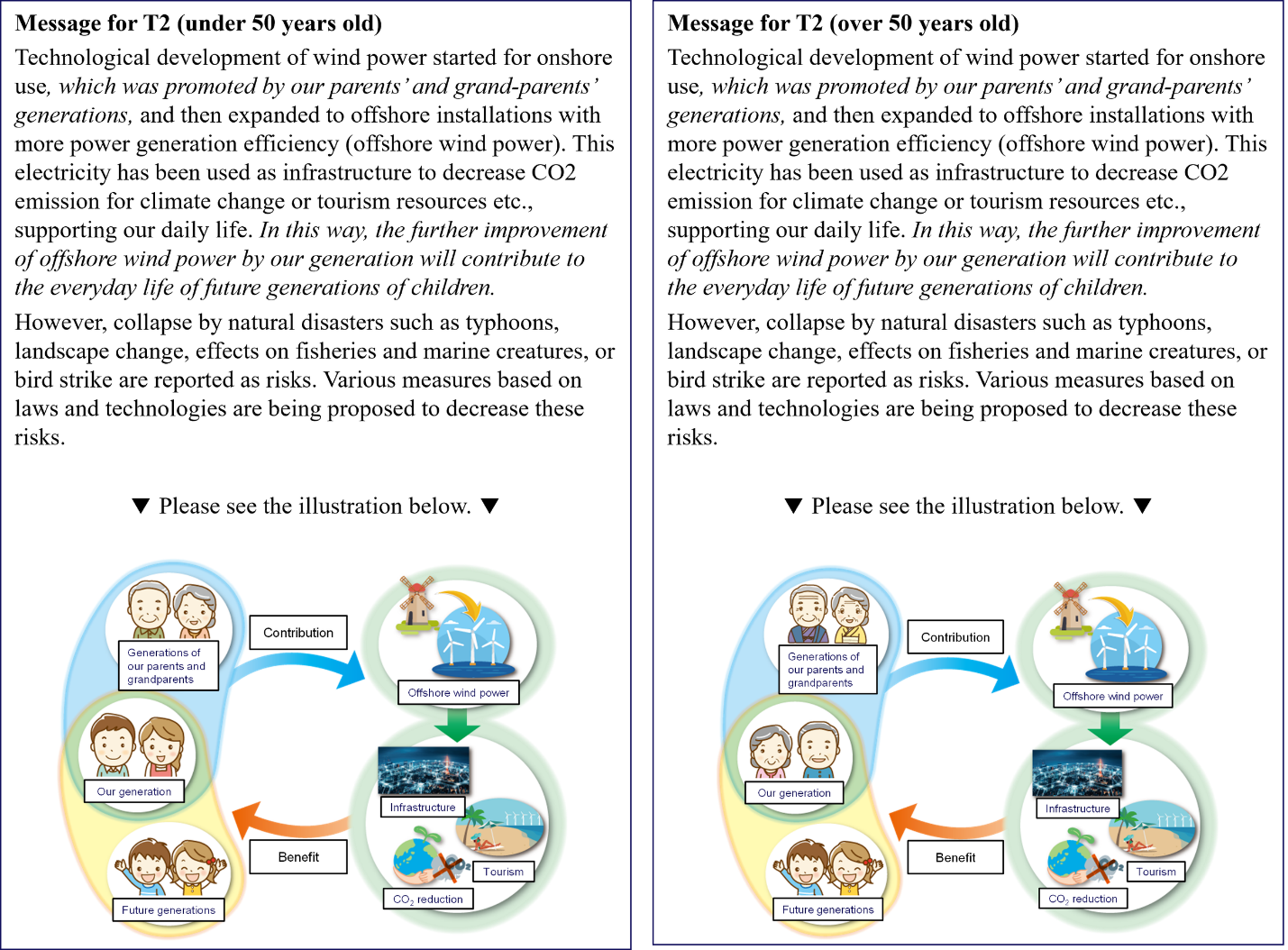


**Fig C. Messages Presented to the T2 Group.** The parts of the text in italics are the intervention messages. Based on [Komatsu 2020] under a CC BY license, with permission from Komatsu, 2020. The illustrations are similar to but not identical to [Komatsu 2020].

Fig D shows the top 10 prefectures for the number of respondents who mentioned fisheries. All 10 of these prefectures appeared in Table A in S2 Appendix, which showed prefectures whose number of responses were larger than 200 or the amount of fish caught was larger than 100 kt.


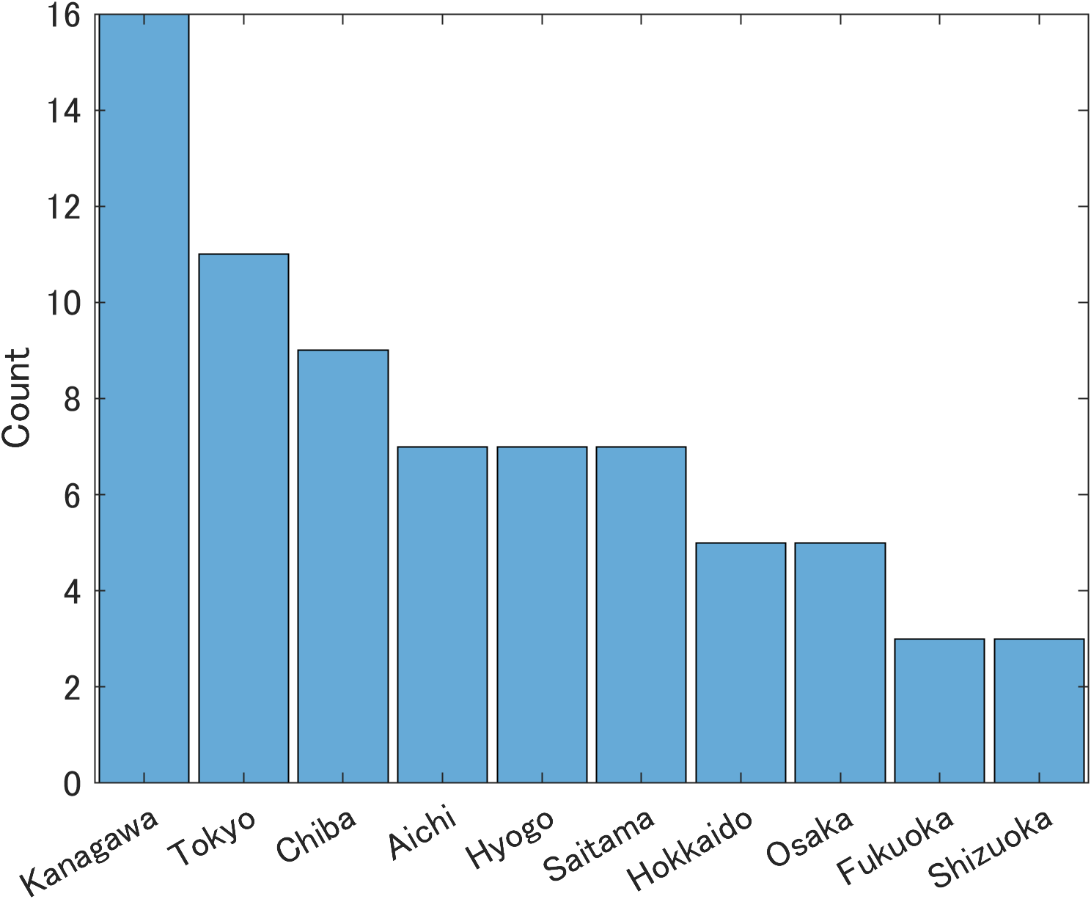


**Fig D. Top 10 Prefectures for Number of Respondents Who Mentioned “Fisheries” in the Open-ended Questionnaire.**
